# Safety of Intraoperative Blood Salvage During Liver Transplantation in Patients With Hepatocellular Carcinoma: A Systematic Review and Meta-analysis

**DOI:** 10.1097/SLA.0000000000005476

**Published:** 2022-07-06

**Authors:** Virginia J. Aijtink, Vera C. Rutten, Beatrice E.M. Meijer, Renate de Jong, John L. Isaac, Wojciech G. Polak, M. Thamara P.R. Perera, Dimitri Sneiders, Hermien Hartog

**Affiliations:** * Department of Surgery, Division of Hepatopancreatobiliary and Transplant Surgery, Erasmus MC Transplant Institute, Erasmus University Medical Center, Rotterdam, The Netherlands; † Department of Anaesthesiology, Erasmus University Medical Center, Rotterdam, The Netherlands; ‡ Department of Anaesthesia, Queen Elizabeth Hospital, University Hospitals Birmingham NHS Foundation Trust, Birmingham, UK; § The Liver Unit, Queen Elizabeth Hospital, University Hospitals Birmingham NHS Foundation Trust, Birmingham, UK; ∥ Institute of Immunology and Immunotherapy, University of Birmingham, Birmingham, UK

**Keywords:** liver transplantation, hepatocellular carcinoma (HCC), intraoperative blood salvage (IBS)

## Abstract

**Objective:**

The effects of intraoperative blood salvage (IBS) on time to tumor recurrence, disease-free survival and overall survival in hepatocellular carcinoma (HCC) patients undergoing liver transplantation were assessed to evaluate the safety of IBS.

**Background:**

IBS is highly effective to reduce the use of allogeneic blood transfusion. However, the safety of IBS during liver transplantation for patients with HCC is questioned due to fear of disseminating malignant cells.

**Methods:**

Comprehensive searches through June 2021 were performed in 8 databases. The methodological quality of included studies was assessed using the Robins-I tool. Meta-analysis with the generic inverse variance method was performed to calculate pooled hazard ratios (HRs) for disease-free survival, HCC recurrence and overall survival.

**Results:**

Nine studies were included (n=1997, IBS n=1200, no-IBS n=797). Use of IBS during liver transplantation was not associated with impaired disease-free survival [HR=0.90, 95% confidence interval (CI)=0.66–1.24, *P*=0.53, IBS n=394, no-IBS n=329], not associated with increased HCC recurrence (HR=0.83, 95% CI=0.57–1.23, *P*=0.36, IBS n=537, no-IBS n=382) and not associated with impaired overall survival (HR=1.04, 95% CI=0.79–1.37, *P*=0.76, IBS n=495, no-IBS n=356).

**Conclusions:**

Based on available observational data, use of IBS during liver transplantation in patients with HCC does not result in impaired disease-free survival, increased HCC recurrence or impaired overall survival. Therefore, use of IBS during liver transplantation for HCC patients is a safe procedure.

Autologous blood transfusion or intraoperative blood salvage (IBS) is a widely used and effective method to reduce the need for allogeneic blood transfusion during high-risk surgery including liver transplantation.^1^ Although IBS is extensively used for liver transplantation in patients with nonmalignant diseases, use in hepatocellular carcinoma (HCC) patients remains controversial. This reluctance towards the use of IBS during oncologic surgery is mainly related to fear of dissemination of malignant cells.^2^,^3^

Allogeneic blood transfusion is associated with both perioperative and long-term risks such as increased mortality and tumor recurrence, extended hospital stay, and more postoperative complications.^1^,^4^–^7^ The exact mechanism behind these observations remains a topic of debate. An immunosuppressive effect may play a role. Previous studies showed decreased function of T-lymphocytes and natural killer cells, increased number of T-suppressor cells, and decreased numbers of macrophages and monocytes in relation to allogeneic blood transfusion.^8^,^9^ In contrast, IBS has been associated with the activation of natural killer cells and upregulated cytokine production, resulting in increased immunocompetence.^9^,^10^

Several studies have proven the efficacy and safety of IBS in surgery for various malignant diseases.^11^–^13^ However, reports evaluating the effect of IBS during liver transplantation for HCC are limited. This meta-analysis provides an overview of current data and aims to assess the safety of IBS in a large sample of HCC patients undergoing liver transplantation by evaluating the effect on time to HCC recurrence, disease-free survival and overall survival.

## METHODS

The Preferred Items for Reporting of Systematic Reviews and Meta-analyses (PRISMA) and Meta-analyses Of Observational Studies in Epidemiology (MOOSE) statements were followed.^14^,^15^ Since only aggregated data was analyzed, approval of the institutional review board was not required. This systematic review was registered in the Prospero database (registration number: 42016037067).

### Study Selection

The EMBASE, MEDLINE (OvidSP), Web-of-science, Scopus, Cochrane, PubMed Publisher, Cinahl Ebsco, and Google Scholar databases were searched through June 2021. The full search syntax is added in Supplemental Digital Content Appendix 1 (http://links.lww.com/SLA/D904) and was provided by a biomedical information specialist. Studies were independently screened by 3 authors (V.J.A., V.C.R., B.E.M.M.), first on the title and abstract and subsequently based on the full-text record. Finally, additional manual cross-referencing was performed on included studies. Any disagreement between reviewers were resolved based on mutual consensus. All studies concerning HCC patients who received IBS during liver transplantation, with or without a leukocyte depletion filter, were eligible for inclusion. If multiple articles reported on a similar source population of patients and outcome of interest, potentially resulting in duplicate data, primarily the sample of patients most in accordance with the entire review sample was used. When no preference based on the sampling within a study could be made, the most recently published data on any outcome of interest were included. Studies with a follow-up ≤12 months were excluded as well as pediatric studies, case reports, non-human studies, and studies not written in English. Studies on IBS with preoperatively retrieved blood were excluded.

### Data Extraction and Quality Assessment

Data extraction was performed using a standard extraction table. The methodological quality of included studies was independently assessed by 3 reviewers (V.J.A., V.C.R., B.E.M.M.) with the validated Robins-I tool.^16^ Discrepancies in data extraction or quality assessment were resolved by consensus. Primary outcomes were time to HCC recurrence, defined as local, locoregional, or distant recurrence of HCC, and disease-free survival, defined as time to either mortality or diagnosis of HCC recurrence. Overall survival was included as a secondary outcome. Relevant baseline characteristics were extracted for the IBS and no-IBS group. Extracted baseline characteristics comprised of use of leukocyte depletion filter, volume of allogeneic, and autologous blood transfused, tumor size, number of tumors, and presence of microvascular or macrovascular invasion. Actual numbers and proportions of recurrence, disease-free survival and overall survival were extracted for 1-, 2-, 3-, and 5-year follow-up.

### Statistical Analysis

Statistical analysis was performed with use of “Open Meta-Analyst” (open-source software based on R statistics) and Review Manager (RevMan, version 5.4.1).^17^,^18^ Survival proportions (at 1, 2, 3, and 5 years after transplantation) were pooled with random-effects models. Hazard ratios (HRs) were pooled according to the Generic Inverse Variance Method.^19^ Proportions and HRs were presented with corresponding 95% confidence intervals (CIs). HRs were extracted directly from articles if reported. If not reported, HRs were calculated from presented Kaplan-Meier curves or according to other methods described previously by Tierney et al.^20^ Quantitative data was estimated from the Kaplan-Meier curve with the use of validated digital image correlation software Webplotdigitzer.^21^ If available, HRs corrected for confounders by either matching or multivariable analysis were extracted. HRs not corrected for confounders and HRs corrected for confounders were pooled separately. In one additional analysis reported corrected HRs and uncorrected HRs were pooled together, were preference was given to corrected effect measures when these were available. Heterogeneity was quantified with the *I* ^2^ statistic. A *P* value <0.05 was considered statistically significant.

## RESULTS

### Literature Search Results

**FIGURE 1:**
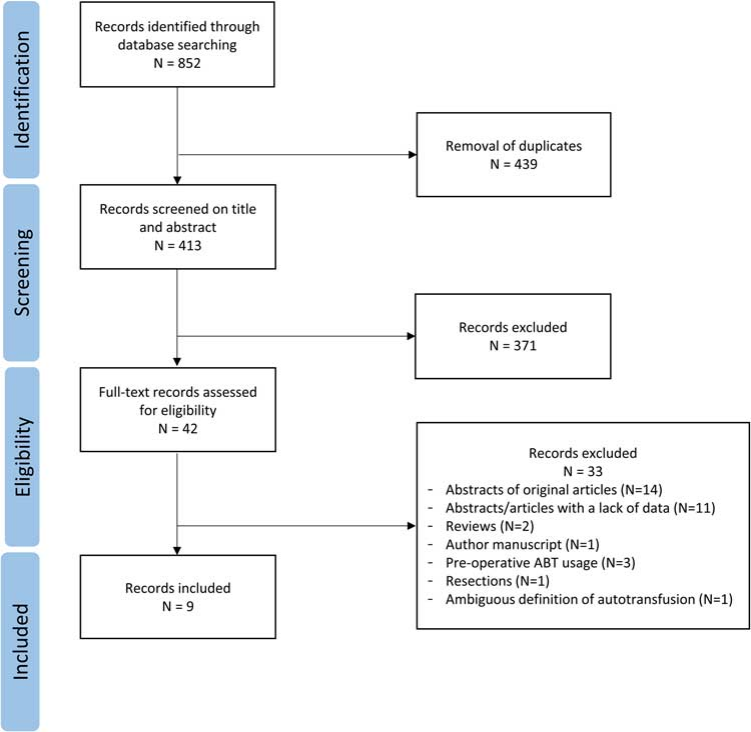
PRISMA flow diagram.

The PRISMA flow diagram is presented in Figure 1. Nine retrospective cohort studies were included, representing 1997 HCC patients undergoing liver transplantation (IBS n=1200, no-IBS n=797).^2^,^22^–^29^ Han et al^25^ and Kwon et al^27^ reported on the same source population of patients while having the same primary outcome (HCC recurrence). Kwon and colleagues included patients with advanced HCC only, while Han and colleagues included a patient cohort that was larger and more comparable to other included studies regarding tumor characteristics. Therefore, for pooled analysis preference was given to inclusion of the data presented by Han and colleagues. Outcome data not presented by the study of Han and colleagues was extracted from Kwon and colleagues. Kim et al^26^ reported on a partly overlapping cohort of the 2 aforementioned studies but included older data. Data from the study of Kim et al^26^ was only used in pooled analysis when the outcomes were not presented by Han and colleagues or Kwon and colleagues. In this way, it was assured that no individual analysis of outcome data contained duplicate data. Quality assessment of included studies is summarized in Supplemental Digital Content Table 1 (http://links.lww.com/SLA/D904). Supplemental Digital Content Table 2 (http://links.lww.com/SLA/D904) provides an overview of inclusion and exclusion criteria and Supplemental Digital Content Table 3 (http://links.lww.com/SLA/D904) provides an overview on the use of locoregional therapies before liver transplantation. Baseline characteristics are presented in Tables 1 and 2. The study by Kwon et al^27^ reported higher percentages of microvascular and macrovascular invasion. Akbulut et al^22^ reported fewer patients within Milan criteria compared with the other studies. Ivanics et al^28^ included only patients who were incidentally diagnosed with HCC on explant histology, resulting in a smaller tumor size, lower number of tumors, and a lower percentage of microvascular invasion. Three studies reported no routine use of a leukocyte depletion filter for IBS.^22^,^24^,^28^ With regard to the leukofiltration technique, one study reported double filtration of salvaged blood,^2^ 3 studies reported single filtration.^25^–^27^

**TABLE 1:** Patient Characteristics of Included Studies

	No. Patients			Allogeneic Blood Transfusion (U)			Follow-up (mo)	
References	IBS	No-IBS	Leukocyte Depletion Filter	IBS	No-IBS	IBS (mL)	IBS	No-IBS
Foltys et al^2^	40	96	Yes	9 (2–22)*	7 (2–40)*	1130 (200–5300)*	38 (1–131)*	29 (0–109)*
Akbulut et al^22^	24	59	No	—	—	—	26±15	18±13
Kim et al^26^	121	109	Yes	4±4	10±18	1590±1487	53 (8–95)*	33 (6–95)*
Han et al,^25^ unmatched	283	114	Yes	3±3	1±3	1391±1490	—	—
Han et al,^25^ matched	222	97	Yes	2±3	1±3	1177±1318	—	—
Araujo et al^23^	122	36	Yes	—	—	—	25 (−)	32 (−)
Pinto et al^24^	122	34	No	2±3	2±2	—	45±33	55±51
Nutu et al,^29^ unmatched	192	186	—	—	—	—	65±32	78±46
Nutu et al,^29^ matched	127	127	—	3±3	1±2	1075±1014	—	—
Kwon et al,^27^ unmatched	220	129	Yes	2 (0–3)	0 (0–2)	811 (497–1247)	—	—
Kwon et al,^27^ matched	74	74	Yes	0 (0–2)	0 (0–2)	—	—	—
Ivanics et al,^28^ unmatched	76	34	No	5 (3–7)	6 (4–10)	750 (500–1480)	68 (36–93)	71 (17–105)
Ivanics et al,^28^ matched	26	26	No	3 (2–6)	6 (4–10)	550 (400–830)	—	—

Continuous variables are presented as mean±SD or median (IQR).

* The value represents median (range). Discrete variables are presented as absolute number.

— indicates not reported.

**TABLE 2:** Tumor Characteristics of Included Studies

	Largest Tumor Size (mm)		No. Tumors		Microvascular Invasion (%)		Macrovascular Invasion (%)	
References	IBS	No-IBS	IBS	No-IBS	IBS	No-IBS	IBS	No-IBS
Foltys et al^2^	25 (5–60)*	23 (8–105)*	—	—	30	21	—	—
Akbulut et al^22^	—	—	—	—	—	—	8	5
Kim et al^26^	2±2	3±2	3±2	2±2	36	13	—	—
Han et al,^25^ unmatched	—	—	—	—	35	41	—	—
Han et al,^25^ matched	—	—	—	—	34	38	—	—
Araujo et al^23^	25 (19–30)	25 (18–35)	2 (1–3)	2 (1–3)	—	—	—	—
Pinto et al^24^	—	—	—	—	—	—	—	—
Nutu et al,^29^ unmatched	—	—	2±2	2±2	54	50	7	8
Nutu et al,^29^ matched	—	—	2±2	2±2	52	52	6	7
Kwon et al,^27^ unmatched	—	—	—	—	66	57	13	13
Kwon et al,^27^ matched	—	—	—	—	60	57	14	15
Ivanics et al,^28^ unmatched	12 (8–16)	15 (10–18)	1 (1–2)	1 (1–2)	8	9	—	—
Ivanics et al,^28^ matched	12 (7–16)	15 (12–20)	1 (1–2)	1 (1–2)	4	12	—	—

Continuous variables are presented as mean±SD or median (IQR).

* The value represents median (range). Discrete variables are presented as absolute number.

— indicates not reported.

### Allogeneic and Autologous Transfusion

The mean or median (as reported) volume of allogeneic blood transfused ranged from 0 to 10 units of red blood cell concentrate and seemed substantially higher in the series by Foltys et al^2^ and Kim et al^25^ (Table 1). The mean or median (as reported) volume of autologous blood transfused ranged between 550 and 1590 mL which would correspond to ∼2 to 6 units of allogeneic red blood cell concentrate.

### Disease-free Survival

**FIGURE 2:**
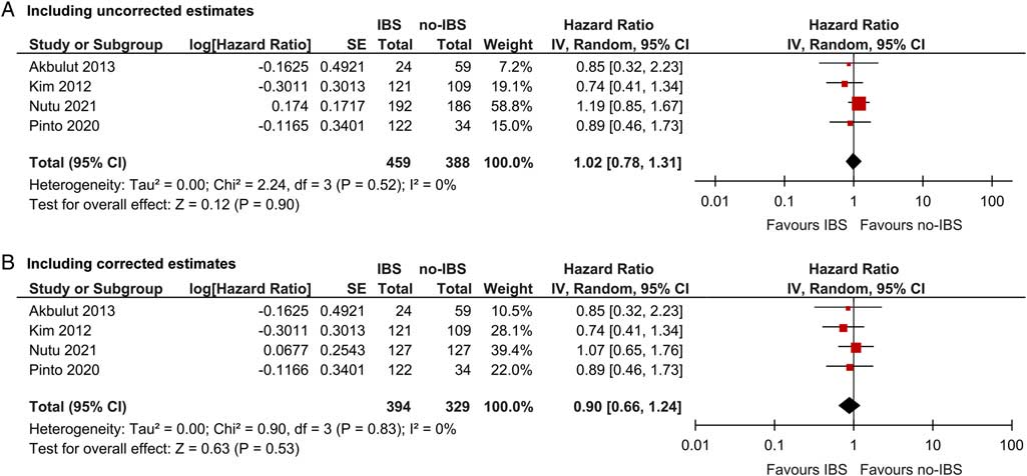
Forest plots representing disease-free survival. A, Forest plot includes uncorrected estimates only. B, Forest plot includes corrected estimates (propensity score matching or multivariable cox regression) if available.

Actual disease-free survival rates in each study are summarized in Table 3. Reported 5-year disease-free survival ranged between 64% and 83% in patients who received IBS and 64% and 77% in patients without IBS. Estimated pooled proportions of patients alive without HCC after 1, 3, and 5 years were 87%, 74%, and 71% for the IBS group and 87%, 71%, and 71% for the no-IBS group. One study used propensity score matching to ensure balanced groups and reported corrected HRs,^29^ whereas 3 studies only reported uncorrected HRs, therefore no separate analysis was performed with corrected effect estimates only.^22^,^24^,^26^ IBS appeared not associated with impaired disease-free survival based on uncorrected estimates (HR=1.02, 95% CI=0.78–1.31, *P*=0.90, IBS n=459, no-IBS n=388, Fig. 2A). When including the corrected effect estimate from the study by Nutu and colleagues in this analysis these association remained similar (HR=0.90, 95% CI=0.66–1.24, *P*=0.53, IBS n=394, no-IBS n=329, Fig. 2B). No significant between-study heterogeneity was present.

**TABLE 3:** Disease-free Survival in Included Studies

			Disease-free Survival (%)			
References	n	IBS or No-IBS	1 y	2 y	3 y	5 y
Kim et al^26^	121	IBS	91	*84*	83	83
	109	No-IBS	85	*82*	79	77
Akbulut et al^22^	24	IBS	*82*	*72*	*72*	—
	59	No-IBS	*84*	*75*	*60*	—
Pinto et al^24^	122	IBS	82	*77*	71	67
	34	No-IBS	85	*81*	64	64
Nutu et al^29^	192	IBS	*86*	*75*	*69*	*64*
	186	No-IBS	*89*	*82*	*75*	*68*
IBS						
Pooled proportion			87	78	74	71
95% CI			82–91	73–83	66–81	60–83
*I* ^2^			37	42	68	88
*P* (for *I* ^2^)			0.193	0.161	0.026	<0.001
No-IBS						
Pooled proportion			87	81	71	71
95% CI			84–90	77–85	63–79	64–78
*I* ^2^			0	0	64	47
*P* (for *I* ^2^)			0.778	0.701	0.041	0.153

— indicates not reported.

Percentages in italic were not reported but derived from charts.

### Time to HCC Recurrence

**FIGURE 3:**
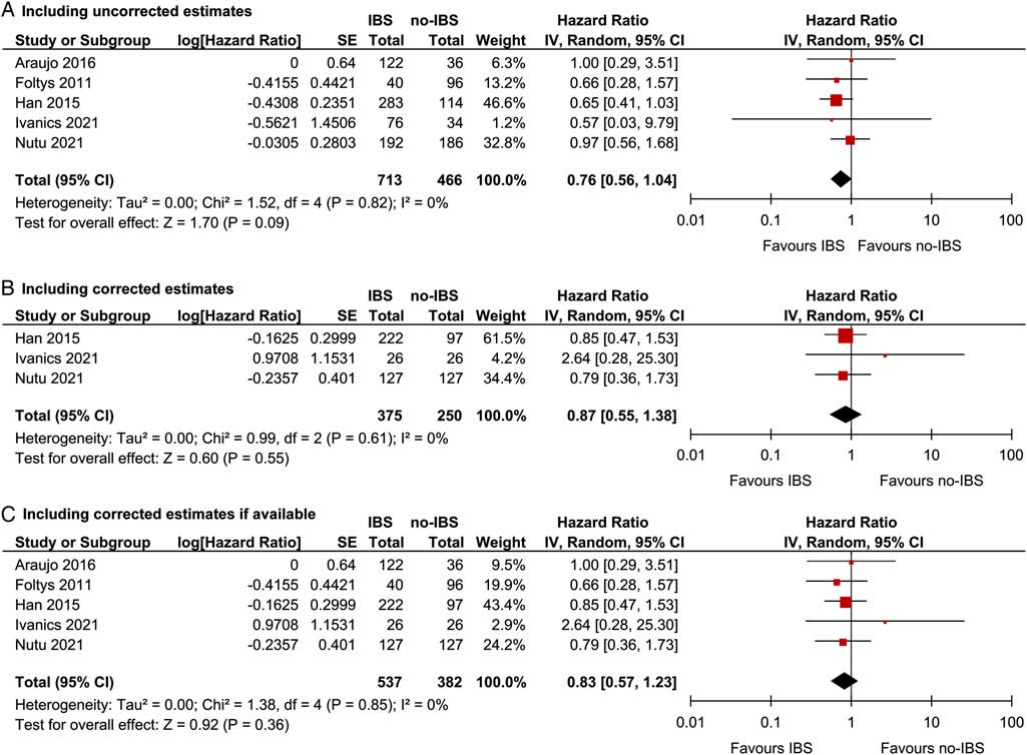
Forest plots representing risk for HCC recurrence. A, Forest plot includes uncorrected estimates only. B, Forest plot includes corrected estimates (propensity score matching or multivariable cox regression) only. C, Forest plot includes uncorrected estimates and corrected estimates (propensity score matching or multivariable cox regression) if available.

Actual HCC recurrence rates are summarized in Table 4. Reported 5-year HCC recurrence rates ranged between 2% and 36% in patients who received IBS compared with 3% and 40% in patients without IBS. The estimated pooled proportion of patients with HCC recurrence after 1, 3, and 5 years were 5%, 10% and 14% for the IBS group and 6%, 13%, and 20% for the no-IBS group. Four studies used propensity score matching and reported corrected HRs,^25^,^27^–^29^ whereas 2 studies reported uncorrected HRs only.^2^,^23^ Figure 3 shows that IBS appeared not associated with HCC recurrence when including uncorrected estimates (HR=0.76, 95% CI=0.56–1.04, *P*=0.09, IBS n=713, no-IBS n=466, Fig. 3A), corrected estimates only (HR=0.87, 95% CI=0.55–1.38, *P*=0.55, IBS n=375, no-IBS n=250, Fig. 3B) or a combination of the 2 (HR=0.83, 95% CI=0.57–1.23, *P*=0.36, IBS n=537, no-IBS n=382, Fig. 3C). No significant between-study heterogeneity was present.

**TABLE 4:** HCC Recurrence in Included Studies

			HCC Recurrence (%)			
References	n	IBS or No-IBS	1 y	2 y	3 y	5 y
Foltys et al^2^	40	IBS	*11*	*11*	*14*	*14*
	96	No-IBS	*10*	*14*	*22*	*31*
Han et al^25^	283	IBS	9	14	—	19
	114	No-IBS	12	23	—	27
Araujo et al^23^	122	IBS	*5*	*9*	*10*	*16*
	36	No-IBS	*1*	*9*	*15*	*22*
Nutu et al^29^	192	IBS	*3*	*11*	*14*	*16*
	186	No-IBS	*6*	*11*	*14*	*17*
Kwon et al^27^	220	IBS	21	27	*32*	36
	129	No-IBS	24	36	*40*	40
Ivanics et al^28^	76	IBS	0	*2*	2	2
	34	No-IBS	0	*0*	3	3
IBS						
Pooled proportion			5	9	10	14
95% CI			1–8	5–14	3–16	6–21
*I* ^2^			82	79	82	90
*P* (for *I* ^2^)			<0.001	<0.001	<0.001	<0.001
No-IBS						
Pooled proportion			6	11	13	20
95% CI			2–10	4–18	5–21	10–30
*I* ^2^			75	86	81	90
*P* (for *I* ^2^)			0.003	<0.001	<0.001	<0.001

— indicates not reported.

Percentages in italic were not reported but derived from charts.

### Overall Survival

Overall survival rates in each study are summarized in Supplemental Digital Content Table 4 (http://links.lww.com/SLA/D904). The estimated pooled proportion of patients alive after 1-, 3-, and 5-year survival were 85%, 75%, and 69% for the IBS group and 88%, 74%, and 72% for the no-IBS group. Four studies used propensity score matching and reported corrected HRs,^23^,^27^–^29^ whereas 2 studies reported uncorrected HRs only.^22^,^24^ As shown in Supplemental Digital Content Figure 1 (http://links.lww.com/SLA/D904), IBS appeared not associated with impaired overall survival when pooling uncorrected estimates (HR=1.02, 95% CI=0.81–1.27, *P*=0.87, IBS n=756, no-IBS n=478, Supplemental Digital Content Fig. 1a, http://links.lww.com/SLA/D904) nor when pooling corrected estimates (HR=1.07, 95% CI=0.70–1.62, *P*=0.75, IBS n=349, no-IBS n=263, Supplemental Digital Content Fig. 1b, http://links.lww.com/SLA/D904) or a combination of the 2 (HR=1.04, 95% CI=0.79–1.37, *P*=0.76, IBS n=495, no-IBS n=356, Supplemental Digital Content Fig.1c, Supplemental Digital Content 1, http://links.lww.com/SLA/D904). No significant between-study heterogeneity was present.

## DISCUSSION

This study aimed to assess the effect of IBS during liver transplantation for HCC patients on disease-free survival, HCC recurrence and overall survival after liver transplantation. None of the included studies reported a significant association between the use of IBS and impaired disease-free survival, increased recurrence rates or impaired overall survival. Pooled results unambiguously indicate that the use of IBS during liver transplantation resulted in equal posttransplant recurrence rates and overall survival as compared with patients who did not receive IBS.

Use of IBS during oncologic surgery remains controversial. In theory, IBS may cause the dissemination of malignant cells in the systemic circulation.^30^ However, the majority of previous studies on the use of IBS for oncologic surgery did not find an association between IBS and recurrence of malignant disease. Waters et al^11^ evaluated the use of IBS in oncologic surgery and demonstrated that the majority of studies reported no difference in recurrence rates, whereas some even reported lower recurrence rates after the use of IBS. In subgroup analysis on prostate cancer and colorectal cancer, no significant differences in recurrence between the IBS and no-IBS groups were reported.^11^ These results are concordant with the present analysis.

Previous studies also suggested that IBS may not be a source of disseminated tumor cells. Hansen et al^31^ provided a case series (n=61) in which 26% of the patients had circulating tumor cells in a venous blood sample after oncologic surgery, not caused by the use of IBS. Thereby, Kumar et al^13^ suggested that morphologic changes and physical traumatism on neoplastic cells due to the salvage process alone lead to loss of viability. The danger of small numbers of circulating malignant cells, in the context of IBS, may be questioned as the metastatic process is very inefficient due to the regulation of malignant-cell growth in secondary sites.^32^ As an example, an in vivo experiment where cultured melanoma cells were injected into the mesenteric veins of healthy mice, only 2% of the cells had the capacity to form micro metastasis.^33^ Therefore, the risk of disseminated malignant cells giving rise to metastasis through the use of IBS could be very low on a theoretical basis.

The use of a leukocyte depletion filter proved to be effective in preventing the dissemination of malignant cells.^34^–^36^ IBS in combination with a leukocyte depletion filter has been reported to cause lethal morphologic damage to the majority of circulating malignant cells. Nearly all cells may show morphologic damage, while 62% of circulating malignant cells present lethal damage.^37^ Nevertheless, uncertainty remains on the need of a single or double-filtered leukoreduction, or need for a leukocyte reduction filter at all.^38^ Double-filtered leukoreduction delays the preparation of IBS and possibly enlarges the need for allogeneic transfusion.^27^ None of the included studies, without or with the use of single-filtered or double-filtered leukoreduction showed a significant association with HCC recurrence. In case reports, severe hypotension after blood salvage with the use of a leukocyte depletion filter has been reported during 2 different cesarean sections.^39^ Although this complication, if related, is likely very rare, the necessity of leukofiltration during blood salvage for oncological surgery remains likewise unclear.

The included studies indicate that the IBS group received almost equal volumes of allogeneic blood transfusion compared with the no-IBS group. This is likely a result of confounding by indication, because patients receiving IBS may be more likely to suffer from a higher degree of blood loss. It is conceivable that use of IBS would still reduce the need for allogeneic blood transfusion in these patients.^40^ Considering mean reported volumes of autologous blood transfused, use of IBS may have resulted in saving on average 2 to 6 U of red blood cell concentrate, leading to a more cost-effective procedure.^41^

In contrast to the use of IBS, allogeneic blood transfusion has been associated with multiple adverse effects including tumor recurrence.^1^,^4^–^7^,^42^ Therefore, we may hypothesize that IBS could in fact be a safe alternative for HCC liver transplant patients to reduce the need for allogeneic blood transfusion and prevent related complications.

### Limitations

This meta-analysis has several limitations. Included studies were of moderate methodological quality and of observational design. Nevertheless, based on qualitative examination of reported baseline characteristics the IBS and no-IBS groups appear reasonably comparable. Moreover, reported results of included studies appear consistent. Nevertheless, we cannot exclude that clinical selection might have influenced results. The decision to use (or not use) IBS in HCC liver transplant patients may be related to tumor load as well as intraoperative blood loss, factors related to respectively tumor recurrence and overall survival. These factors could be related to the intervention and outcomes and qualify as confounders. A number of studies adequately corrected tumor-related factors, results of those studies were provided separately. In addition, we did not identify tumor load was consistently reported to be lower in patients who received IBS. The series reported by Ivanics et al^28^ may substantially differ from other studies, as only patients with incidental HCC on the liver explant were included. Kwon et al,^27^ on the other hand, only included patients with advanced HCC. Akbulut et al^22^ included more patients beyond Milan and UCSF criteria. However, despite including patients with different degrees of tumor load, the 3 aforementioned studies did not report substantially different results. Increased blood loss is logically related to increased use of IBS and also related to impaired overall survival (confounding by indication). Since overall survival in IBS patients was not impaired, this is not of concern and supports the safety of IBS. No sufficient data is available to assess the need for a leukocyte reduction filter, regardless of its use, no study reported a significant association between the use of IBS and subsequent HCC recurrence. Not all articles provided accurate information on follow-up, numbers at risk at specific time points, and numbers of censored cases. Therefore, methods for data extraction as previously described by Tierney et al^20^ were adapted. The majority of studies does not further define the recurrence site, therefore no distinction between extrahepatic and intrahepatic recurrence could be made.

## CONCLUSIONS

Based on current data, use of IBS during liver transplantation for patients with HCC was not associated with an increased risk for either HCC recurrence, impaired disease-free survival or overall survival. IBS may be considered a safe alternative to reduce the need for allogeneic blood transfusion in patients with HCC undergoing liver transplantation.

## Supplementary Material

SUPPLEMENTARY MATERIAL
