# Supplementary material for: Safety of Intraoperative Blood Salvage During Liver Transplantation in Patients With Hepatocellular Carcinoma: A Systematic Review and Meta-analysis
Source: Ann Surg. 2022 Jul 6;276(2):239–45. doi: 10.1097/SLA.0000000000005476 (PMC9259047; doi:10.1097/SLA.0000000000005476)
Supplement: SUPPLEMENTARY MATERIAL [file sla-276-0239-s001.docx]

**Supplements**

**Supplemental Table 1. Quality Assessment**

|  |  | **Study** | | | | | | | | | |
| --- | --- | --- | --- | --- | --- | --- | --- | --- | --- | --- | --- |
|  |  | **Akbulut (2013)** | **Araujo (2016)** | **Foltys (2011)** | **Han (2015)** | **Ivanics (2021)** | **Kim (2013)** | **Kwon (2021)** | **Nutu (2021)** | **Pinto (2020)** |  |
| **Risk of bias domains*** | Confounding | Serious | Moderate | Moderate | Low | Low | Moderate | Low | Low | Moderate |  |
|  | Selection of participants | Low | Low | Low | Low | Serious | Low | Low | Low | Low |  |
|  | Classification of interventions | Low | Low | Low | Low | Low | Low | Low | Low | Low |  |
|  | Deviations from intended interventions | Low | Low | Moderate | Low | Low | Low | Low | Moderate | Low |  |
|  | Missing data | Low | - | Low | Low | Low | Moderate | Low | Moderate | Low |  |
|  | Measurement of outcomes | Low | Low | Low | Low | Low | Low | Low | Low | Low |  |
|  | Selection of the reported result | Low | Moderate | Low | Low | Low | Low | Low | Low | Low |  |
|  | Overall | Serious | Moderate | Moderate | Low | Serious | Moderate | Low | Moderate | Moderate |  |

-: not reported. * Risk of bias domains as described in the Robins-I tool.

**Supplemental Table 2. definition of study populations**

|  | Definition of studied population | | | |
| --- | --- | --- | --- | --- |
|  | **Inclusion of patients with incidental HCC diagnosis on explant** | **Inclusion of patients with Advanced/outside criteria HCC** | **Inclusion of patients undergoing LDLT*** | **Other relevant exclusion criteria** |
| Foltys (2011) | Yes | Yes | No | Complete tumor necrosis |
| Akbulut (2013) | Yes | Yes | Yes | Mortality < 3 months (non-HCC cause) |
| Kim (2013) | No | Yes | Only | (1) Mortality < 1 month (non-HCC cause)  (2) No need for blood transfusion |
| Han (2015) | No | Yes | Only | Macrovascular tumor invasion |
| Araujo (2016) | No | No | No | None |
| Pinto (2020) | Unknown | Yes | No | None |
| Nutu (2021) | No | Yes | No | None |
| Kwon (2021) | No | Only | Only | None |
| Ivanics (2021) | Only | No | Yes | None |

*When not reported specifically studies were assumed to include deceased donor liver transplant recipients. HCC: hepatocellular carcinoma, LDLT: living donor liver transplantation.

**Supplemental Table 3. Reported bridging/downstaging therapies**

| Study | Bridging/ downstaging LRT n (%) | | LRT specified | |
| --- | --- | --- | --- | --- |
|  | **IBS** | **No-IBS** | **IBS** | **No-IBS** |
| Foltys (2011) | 15/40 (38) | 76/96 (79) | TACE: 15 | TACE: 76 |
| Akbulut (2013) | - | - | - | - |
| Kim (2013) | - | - | - | - |
| Han (2015) | Unmatched  - | - | - | - |
|  | Matched  149/222 (67) | 70/97 (72) | TACE, RFA, resection* | TACE, RFA, resection* |
| Araujo (2016) | 61/122 (50) | 19/36 (53) | RFA: 4 TACE: 69 Ethanol: 7 | RFA: 3 TACE: 53 Ethanol: 5 |
| Pinto (2020) | 90/122 (74) | 23/34 (68) | TACE, RFA, ethanol* | TACE, RFA, ethanol* |
| Nutu (2021) | Unmatched  88/192 (46) | 105/186 (56) | - | - |
|  | Matched  66/127 (52) | 64/ 127 (50) | - | - |
| Kwon (2021) | Unmatched 215/220 (98) | 128/129 (99) | - | - |
|  | Matched 73/74 (99) | 73/74 (99) | - | - |
| Ivanics (2021) | None | None | None | None |

* Numbers not provided. LRT: locoregional therapy, IBS: intra-operative blood salvage, TACE: trans-arterial chemo embolization, RFA: radio-frequency ablation, ethanol: ethanol injection, - not reported.

**Supplemental Table 4. Overall survival in included studies**

|  | | |  |  | **Overall survival (%)** | | | |
| --- | --- | --- | --- | --- | --- | --- | --- | --- |
| **Study** |  | | **n** | **IBS or no-IBS** | **1 year** | **2 year** | **3 year** | **5 year** |
| **Akbulut (2013)** |  | | 24 | *IBS* | *70* | *62* | *62* | - |
|  |  | | 59 | *No-IBS* | *77* | *69* | *58* | - |
| **Araujo (2016)** |  | | 122 | *IBS* | *73* | *68* | *68* | *61* |
|  |  | | 36 | *No-IBS* | *78* | *75* | *72* | *66* |
| **Pinto (2020)** |  | | 122 | *IBS* | 84 | *80* | 77 | 68 |
|  |  | | 34 | *No-IBS* | 85 | *79* | 72 | 68 |
| **Nutu (2021)** |  | | 192 | *IBS* | *87* | *79* | *71* | *64* |
|  |  | | 186 | *No-IBS* | *91* | *85* | *76* | *70* |
| **Kwon (2021)** |  | | 220 | *IBS* | 90 | 83 | *76* | 71 |
|  |  | | 129 | *No-IBS* | 88 | 77 | *70* | 65 |
| **Ivanics (2021)** |  | | 76 | *IBS* | *96* | *90* | *88* | *83* |
|  |  | | 34 | *No-IBS* | *97* | *94* | *91* | *88* |
| **IBS** | |  |  |  |  |  |  |  |
| **Pooled proportion** | |  |  |  | 85 | 79 | 75 | 69 |
| **95% CI** |  | |  |  | 79-92 | 73-85 | 69-81 | 63-76 |
| **I^2^** |  | |  |  | 85 | 75 | 73 | 76 |
| **P (for I^2^)** |  | |  |  | <0.001 | 0.001 | 0.002 | 0.003 |
| **No-IBS** |  | |  |  |  |  |  |  |
| **Pooled proportion** |  | |  |  | 88 | 81 | 74 | 72 |
| **95% CI** |  | |  |  | 82-93 | 74-88 | 66-82 | 64-80 |
| **I^2^** |  | |  |  | 69 | 71 | 75 | 67 |
| **P (for I^2^)** |  | |  |  | 0.006 | 0.004 | <0.001 | 0.015 |

IBS: intraoperative blood salvage, -: not reported. CI: confidence interval. Percentages in italic were not reported but derived from charts.

**Supplemental Figure 1: Forest plots representing overall survival**


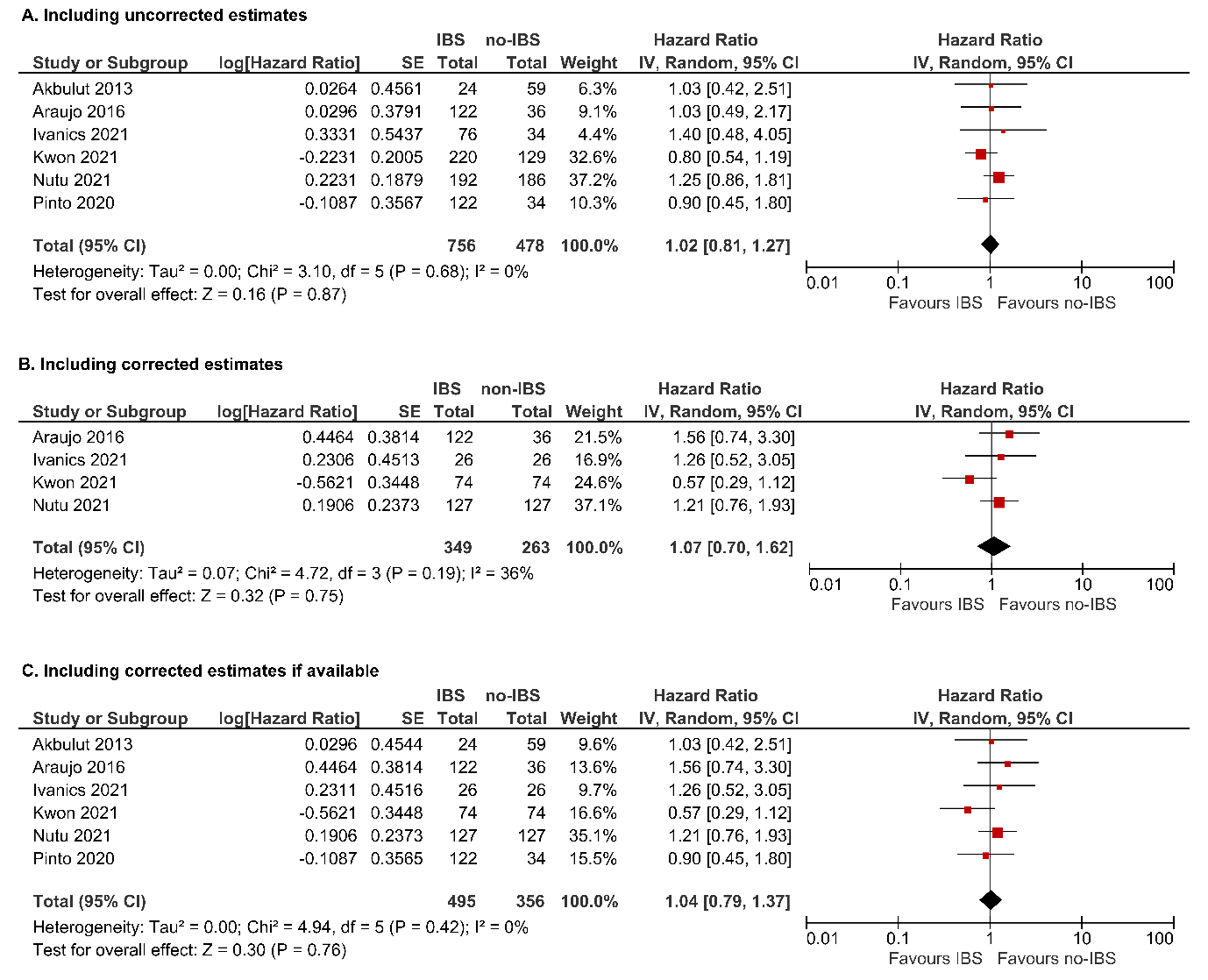


*A) Forest plot includes uncorrected estimates only*

*B) Forest plot includes corrected estimates (propensity score matching or multivariable cox regression) only*

*C) Forest plot includes uncorrected estimates and corrected estimates (propensity score matching or multivariable cox regression) if available*

**Appendix 1 Search strategy***Date of search: June 22, 2021*

**Embase.com 219**

('liver cell carcinoma'/exp OR 'liver tumor'/de OR 'liver cancer'/de OR (((liver OR hepat*) NEAR/3 (carcinom* OR malignan* OR cancer* OR tumo* OR neoplas*)) OR hepatocarcinom* OR hepatom* OR hcc):ab,ti,kw) AND ('blood autotransfusion'/exp OR 'blood salvage'/exp OR 'autotransfusion unit'/de OR ('autotransplantation'/de AND 'blood transfusion'/exp) OR (((blood OR erythrocyt* OR hemo* OR haemo*) NEAR/3 (autotransfusion* OR autologous)) OR autohemotransfus* OR autohaemotransfus* OR autotransfus* OR ((cell OR blood OR erythrocyt*) NEAR/3 (save* OR salvage*))):ab,ti,kw) AND [english]/lim

**Medline ovid 140**

("Carcinoma, Hepatocellular"/ OR "Liver Neoplasms"/ OR (((liver OR hepat*) ADJ3 (carcinom* OR malignan* OR cancer* OR tumo* OR neoplas*)) OR hepatocarcinom* OR hepatom* OR hcc).ab,ti,kf.) AND ("Blood Transfusion, Autologous"/ OR "Operative Blood Salvage"/ OR ("Transplantation, Autologous"/ AND ("Blood Transfusion"/ OR "Blood Component Transfusion"/ OR "Erythrocyte Transfusion"/)) OR (((blood OR erythrocyt* OR hemo* OR haemo*) ADJ3 (autotransfusion* OR autologous)) OR autohemotransfus* OR autohaemotransfus* OR autotransfus* OR ((cell OR blood OR erythrocyt*) ADJ3 (save* OR salvage*))).ab,ti,kf.) AND english.la.

**Cochrane 10**

((((liver OR hepat*) NEAR/3 (carcinom* OR malignan* OR cancer* OR tumo* OR neoplas*)) OR hepatocarcinom* OR hepatom* OR hcc):ab,ti) AND ((((blood OR erythrocyt* OR hemo* OR haemo*) NEAR/3 (autotransfusion* OR autologous)) OR autohemotransfus* OR autohaemotransfus* OR autotransfus* OR ((cell OR blood OR erythrocyt*) NEAR/3 (save* OR salvage*))):ab,ti,kw)

**Web of science 159**

TS=(((((liver OR hepat*) NEAR/2 (carcinom* OR malignan* OR cancer* OR tumo* OR neoplas*)) OR hepatocarcinom* OR hepatom* OR hcc)) AND ((((blood OR erythrocyt* OR hemo* OR haemo*) NEAR/2 (autotransfusion* OR autologous)) OR autohemotransfus* OR autohaemotransfus* OR autotransfus* OR ((cell OR blood OR erythrocyt*) NEAR/2 (save* OR salvage*)))) ) AND LA=(english)

**Scopus 200**

TITLE-ABS-KEY(((((liver OR hepat*) W/2 (carcinom* OR malignan* OR cancer* OR tumo* OR neoplas*)) OR hepatocarcinom* OR hepatom* OR hcc)) AND ((((blood OR erythrocyt* OR hemo* OR haemo*) W/2 (autotransfusion* OR autologous)) OR autohemotransfus* OR autohaemotransfus* OR autotransfus* OR ((cell OR blood OR erythrocyt*) W/2 (save* OR salvage*)))) ) AND LANGUAGE (english)

**Cinahl Ebsco 24**

(MH "Carcinoma, Hepatocellular" OR MH "Liver Neoplasms" OR TI (((liver OR hepat*) N2 (carcinom* OR malignan* OR cancer* OR tumo* OR neoplas*)) OR hepatocarcinom* OR hepatom* OR hcc) OR AB (((liver OR hepat*) N2 (carcinom* OR malignan* OR cancer* OR tumo* OR neoplas*)) OR hepatocarcinom* OR hepatom* OR hcc)) AND (MH "Blood Transfusion, Autologous" OR MH "Blood Salvage" OR (MH "Autografts" AND (MH "Blood Transfusion" OR MH "Erythrocyte Transfusion")) OR TI (((blood OR erythrocyt* OR hemo* OR haemo*) N2 (autotransfusion* OR autologous)) OR autohemotransfus* OR autohaemotransfus* OR autotransfus* OR ((cell OR blood OR erythrocyt*) N2 (save* OR salvage*))) OR AB (((blood OR erythrocyt* OR hemo* OR haemo*) N2 (autotransfusion* OR autologous)) OR autohemotransfus* OR autohaemotransfus* OR autotransfus* OR ((cell OR blood OR erythrocyt*) N2 (save* OR salvage*)))) AND LA (english)

**Pubmed publisher NA**

("Carcinoma, Hepatocellular"[mh] OR "Liver Neoplasms"[mh] OR (((liver[tiab] OR hepat*[tiab]) AND (carcinom*[tiab] OR malignan*[tiab] OR cancer*[tiab] OR tumo*[tiab] OR neoplas*[tiab])) OR hepatocarcinom*[tiab] OR hepatom*[tiab] OR hcc[tiab])) AND ("Blood Transfusion, Autologous"[mh] OR "Operative Blood Salvage"[mh] OR ("Transplantation, Autologous"[mh] AND ("Blood Transfusion"[mh] OR "Blood Component Transfusion"[mh] OR "Erythrocyte Transfusion"[mh])) OR (((blood[tiab] OR erythrocyt*[tiab] OR hemo*[tiab] OR haemo*[tiab]) AND (autotransfusion*[tiab] OR autologous)) OR autohemotransfus*[tiab] OR autohaemotransfus*[tiab] OR autotransfus*[tiab] OR ((cell[tiab] OR blood[tiab] OR erythrocyt*[tiab]) AND (save*[tiab] OR salvage*[tiab])))) AND english[la] AND publisher[sb]

**Google scholar 100**

"liver|hepatic|hepatocellular carcinoma|malignancy|cancer|tumor|neoplasms"|hepatocarcinoma "cell|blood|erythrocytes saver|salvage"
'liver|hepatic|hepatocellular carcinoma|malignancy|cancer|tumor|neoplasms'|hepatocarcinoma 'cell|blood|erythrocytes saver|salvage'
